# Probabilistic dose distribution from interfractional motion in carbon ion radiation therapy for prostate cancer shows rectum sparing with moderate target coverage degradation

**DOI:** 10.1371/journal.pone.0203289

**Published:** 2018-08-31

**Authors:** Daniel Bridges, Hidemasa Kawamura, Tatsuaki Kanai

**Affiliations:** 1 Gunma University Graduate School of Medicine, Maebashi, Gunma, Japan; 2 Gunma University Heavy Ion Medical Center, Maebashi, Gunma, Japan; 3 Osaka Heavy Ion Therapy Center, Osaka, Osaka, Japan; Massachusetts General Hospital, Harvard Medical School, UNITED STATES

## Abstract

**Purpose:**

This observational study investigates the influence of interfractional motion on clinical target volume (CTV) coverage, planning target volume (PTV) margins, and rectum tissue sparing in carbon ion radiation therapy (CIRT). It reports dose coverage to target structures and organs at risk in the presence of interfractional motion, investigates rectal tissue sparing, and provides recommendations for lowering the rate of toxicity. We also propose probabilistic DVH based on cone-beam computed tomography (CBCT) table shifts from photon therapy for consideration in bone-matching CIRT treatment planning to represent probable dose to our CIRT patient population.

**Methods:**

At Gunma University Hospital intensity-modulated x-ray therapy (IMXT, aka IMRT) prostate cancer patients are positioned on a table which is shifted twice based on CBCT to align bones and then align prostate tissue to isocenter. These shifts thereby contain interfractional motion. A total of 1306 such table shifts from 85 patients were collected. Normal probability distributions were fit to the difference between bone-matching and prostate-matching CBCT-to-planning CT table shifts (*i*.*e*. interfractional motion). Between 2011 and 2016 CIRT prostate patients were treated with three beams to PTV1 (lateral-opposing and anterior) one per day for 9 fractions and two beams for a boost PTV2 (lateral-opposing) one per day for 7 fractions for a prescribed total of 57.6 Gy(RBE) as follows: PTV1 extends the prostate contour by 10/10, 5/10, 6/6 mm in the right/left, posterior/anterior, and superior/inferior directions, respectively, and the proximal seminal vesicles contour by 5 mm superiorly and inferiorly, 3 mm right and left. PTV2 reduces PTV1 posteriorly along a straight line to exclude the rectum and reduces the superior and inferior margins by 6 mm. Probable interfractional motion for 40 patients was simulated using each patient’s own beam data as follows: The previously fit normal probability distributions were randomly sampled 2000 times per patient, and the five beams were shifted and summed with the same relative weighting as in the 16-fraction regimen. The resulting dose distribution was then scaled back down by 16/2000 to match the prescribed number of fractions. We then analyzed the resulting doses to contoured structures.

**Results:**

Probable dose to rectum is substantially less than planned: For example, mean+-standard deviation D2% for planned and probable DVH is 51+-1.9 and 45+-2.4, respectively. Cumulative DVH show mean CTV fraction receiving a given probable dose is less than the mean fraction receiving the corresponding planned dose for doses larger than 52 Gy(RBE), up to 19% less at 57.4 Gy(RBE). Our PTV1 margins generally cover 95% of interfractional motion but seminal vesicles and inferior prostate receive less dose than planned due to insufficient PTV2 margins.

**Conclusion:**

Assuming rigidly shifting interfractional motion around the prostate region and neglecting minor changes in soft tissue stopping power, interfractional motion resulted in target underdosing but better tissue sparing in all cases. Given our low rates of relapse and recurrence, it appears less curative dose is needed than previously thought or else current planning target margins may be excessive: Planning target volumes should be reconsidered with the adoption of dose verification methods. Our probable dose distributions quantify expected dose for future dose verification studies.

## Introduction

In fractionated prostate cancer radiation therapy intrafractional and interfractional motion must both be accounted for in patient treatment. The prostate is known to move up to 2 mm during photon irradiation, [1] and up to 7 mm between fractions. [2] This study concerns interfractional motion. Intrafractional motion is not a problem because carbon beam treatment time is approximately one minute, but the interfractional motion remains a concern. Currently we treat the patient based on bone-matching, removing gas if present in portal x-rays. Accounting for interfractional motion has been a problem in CIRT. In intensity-modulated x-ray therapy cone-beam computed tomography (CBCT) is used to shift the target to the beam’s isocenter to ensure target coverage. We cannot do this because carbon ion beam range depends on stopping power ratio along the beam path: [3] Shifting the patient to keep the target at isocenter can change bony anatomy along the beam path degrading the dose distribution. Therefore we irradiate the planned target volumes without accounting for the target’s shift, and yet our toxicity is remarkably low [4] with more than 90% of patients recurrence-free after 48 months’ followup. [5] This study provides a dosimetric reason for these good treatment results and a method to assess the consequences of interfractional motion during treatment planning. Finally, recently in-room CT has been introduced at the Gunma University Heavy Ion Medical Center (GHMC) and at other carbon beam centers. Our findings suggest further scrutiny of target volume definition is needed by in-room CT dose verification.

## Method

### Institutional review board and data use authorization

This retrospective study was approved by the Gunma University Hospital (GUH) institutional review board and it did not require informed consent.

### Interfractional motion data collection

The foundation for this study is that the interfractional motion of the targets and anterior rectal wall proximal to the prostate is measurable by the recorded Elekta table shift coordinates. In other words, we quantify interfractional prostate motion using the Elekta table shift coordinates of the prostate alignment to IMXT isocenter. To obtain this data, 1306 table shifts collected between April 3rd, 2013 and January 1st, 2016 for 85 prostate patients treated with Elekta IMXT at Gunma University Hospital Elekta were exported using Dr.View/RTiS version 2.9 software (InfoCom). We apply these table shifts to our carbon radiotherapy patient population, thereby assuming that interfractional motion is not affected by our immobilization devices. We justify this assumption by noting that interfractional organ motion is determined by physiological processes. [6] and we do not compress the abdomen in either case. Our bone-matching is within one-millimeter accuracy, [7–9] so residual motion due to bone-displacement is negligible.

Patients are asked to empty their rectum and given a glycerin enema if needed. Patients are asked to void their bladder 30 minutes before treatment to obtain a natural semi-full bladder. They are positioned on a Vac-Loc immobilization bag for the legs and feet, beginning a few centimeters below the gluteus maximus. These patients are aligned on the table according to skin markings and a kilovoltage (kV) CBCT is taken to check for gas. The gas is removed if present. Two sets of table coordinates are determined using Elekta XVI release 4.2.2 b15 (Elekta Limited) and recorded using Dr.View/RTiS.

The standard prostate cancer photon therapy regimen at Gunma University Hospital is 3 Gy per fraction Monday, Wednesday, and Friday for seven weeks (63 Gy). The patient is positioned on the table and skin markings are aligned to in-room lasers. A 120 kV 1038.4 mAs CBCT is then taken of the patient’s pelvis. Bone-matching coordinates $\vec{C_{\text{bone}}}$ are recorded after aligning the patient’s bones to the planning CT via Elekta XVI, and then soft tissue matching coordinates $\vec{C_{\text{soft}\;\text{tissue}}}$ are recorded after manually aligning the prostate to the planning CT.

Interfractional motion $\vec{I}$ was calculated as the difference between table bone-matching coordinate $\vec{C_{\text{bone}}}$ and table CBCT-to-planning-CT soft tissue matching coordinate $\vec{C_{\text{soft}\;\text{tissue}}}$:

$$\vec{I}(x,y,z)=\vec{C_{\text{bone}}}(x,y,z)-\vec{C_{\text{soft}\;\text{tissue}}}(x,y,z).$$

### Interfractional motion probability distributions and CIRT data collection

**Fig 1:**
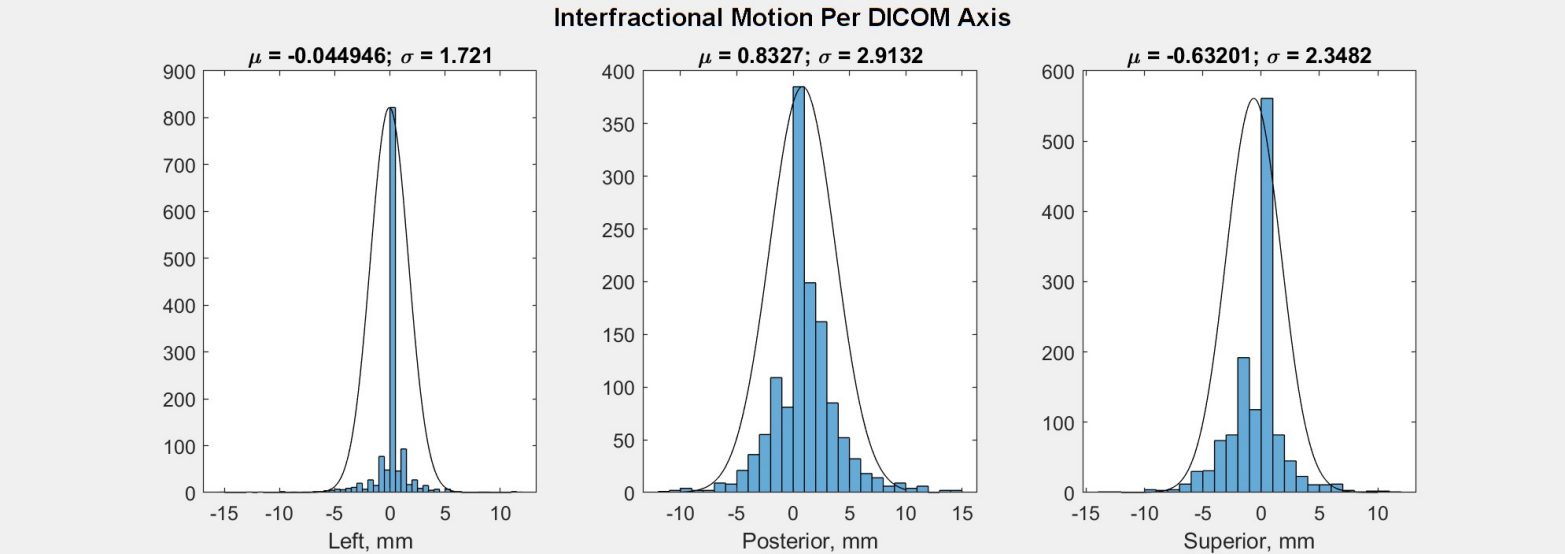
Normal probability distributions fit to interfractional motion per DICOM axis.

Normal probability distributions were fit to this interfractional motion in MATLAB version 9.4.0.813654 (The MathWorks, Inc.) after converting from Elekta table axes to DICOM axes (Fig 1). To apply this probable interfractional motion to carbon radiotherapy patients, 40 prostate CIRT patients’ RTDose and RTPlan DICOM files were exported from MIM Maestro version 6.7.9 (MIM Software Inc.) after anonymization. These patients were randomly selected with the constraints that there be at least ten patients with no toxicity, at least ten patients with genitourinary toxicity, at least ten patients with gastrointestinal toxicity, and that they have received treatment prior to 2016.

### 16-fraction prostate cancer therapy at the Gunma University Heavy Ion Medical Center

#### Prostate therapy PTV margins

Target structures for the CTV include the prostate and proximal one- to two-thirds of the seminal vesicles as indicated by disease. (How the CTV is delineated differs between Japanese institutions.) From these structures the PTV is generated, and range compensators are used to supplement the PTV to account for changes in beam depth per beam direction. The radiation oncologist considers dose to the PTV rather than contouring a CTV as an extension of a GTV.

**Fig 2:**
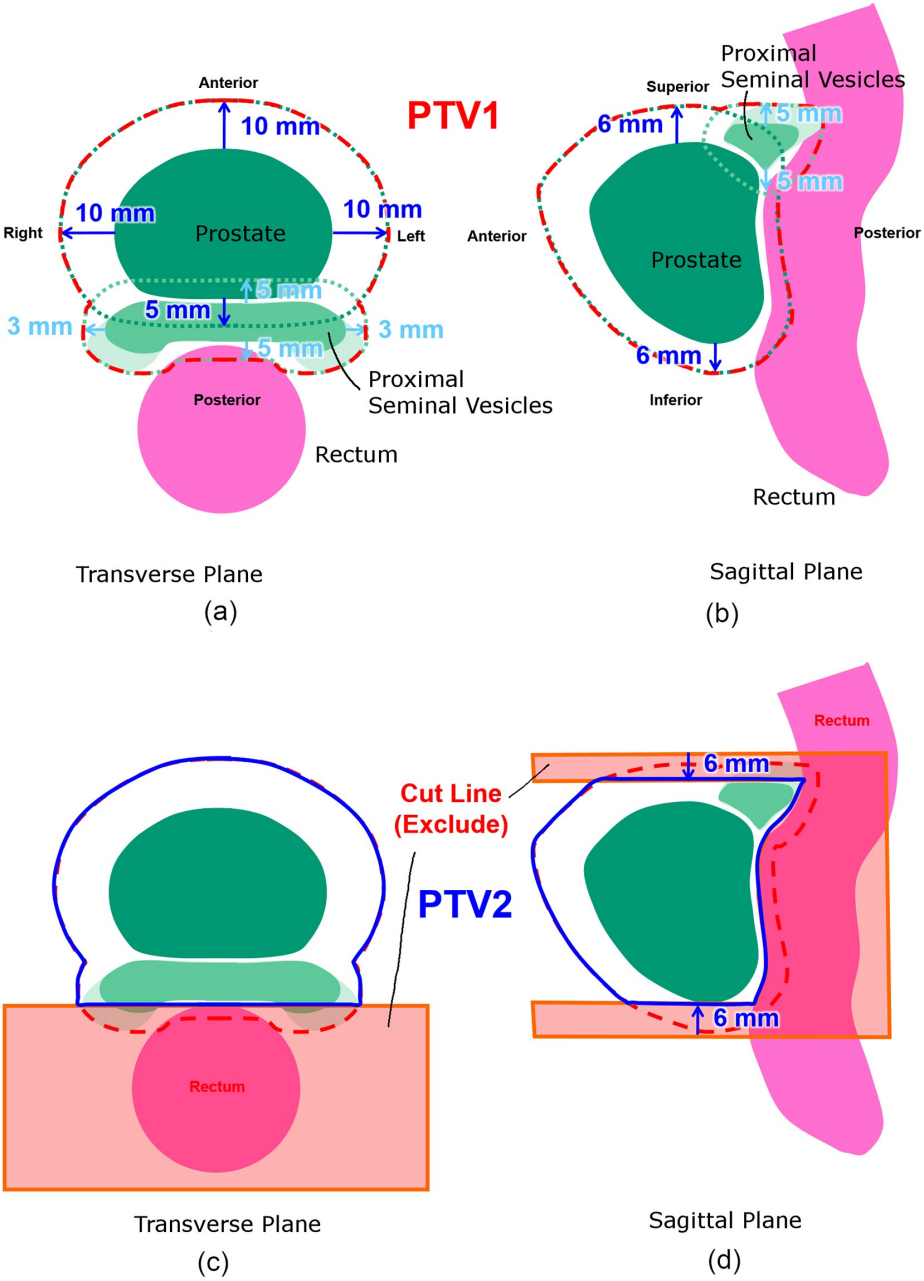
Schematic showing (a,b) PTV1 and (c,d) PTV2 for 16-fraction prostate CIRT at the GHMC. Image revised with permission from the GHMC treatment guidelines.

See Fig 2a and 2b: For our patient sample, PTV1 extends the prostate contour by 10/10, 5/10, 6/6 mm in the right/left, posterior/anterior, and superior/inferior directions, respectively, and the proximal seminal vesicles contour by 5 mm superiorly and inferiorly, 3 mm right and left. See Fig 2c and 2d: PTV2 reduces PTV1 posteriorly along a straight line to exclude the rectum and reduces the superior and inferior margins by 6 mm. Additional information may be found in the published literature. [5]

#### Plan description and beam considerations

Prostate CIRT treatment plans consist of *f* = 16 fractions using *n* = 5 beams (left, right, and anterior targeting PTV1 and left, right targeting PTV2) each with *D*_*i*_ = 3.6 Gy(RBE) prescribed to the normalization point to sum to 57.6 Gy(RBE). Four of the beams are applied three times and one beam (left or right, depending on patient anatomy) is applied four times (let us denote these beam application weights as *w*_*i*_). Three fractions are delivered in the anterior-posterior (AP) direction and the other 13 are delivered laterally. Although rare, oblique fields may replace lateral fields if the patient has metallic hip implants or prosthesis. [7] Patients receive an enema prior to planning CT, simulation, and a 100 mL bladder injection before AP treatment. One beam is delivered daily Tuesday through Friday. Patients in this study were treated during 2011–2015.

We treat based on bony-alignment rather than prostate localization. This minimizes changes in tissue stopping power (i.e. changes to the water-equivalent path length) along the beam path, helping ensure the Bragg peak occurs within the target. Because stopping power ratio is essential for particle beam range, gas is removed if visible on x-ray images. Patients are immobilized using a 3 mm firm thermoplastic shell covering the femur, pelvic bones, and lower abdomen. Additional details concerning patient treatment can be found in the published literature. [8]

As part of routine treatment planning, dose-volume histograms of the patient volume, prostate, proximal seminal vesicles (differing amounts included depending on disease indication), rectum, bladder, PTV1, and PTV2 are considered. The bladder and rectum are regarded as organs at risk. In particular, at the GHMC we commonly compare the rectum DVH to a reference line established from initial clinical trials (using data visualized in Fig 3 of Ishikawa *et al*. [5]) with two standard deviations indicated to estimate the risk of rectum toxicity.

### Most probable blurred dose distribution

We define blurred dose as the expected dose to a target moving an infinitesimal distance $d\vec{r}$ with probability *p* given planned beam doses *D*:

$$B(\vec{r})=∭p(d\vec{r})D(\vec{r}+d\vec{r})d\vec{r}.$$

We say ‘blurred’ because the planned dose distribution is blurred slightly by the interfractional motion. For *f* treatment fractions over *n* beams with interfractional motion *Δr* randomly sampled by the MATLAB probability object a sufficiently large number *N* times, this integration is approximated as

$$B(\vec{r})≅\frac{f}{N}\sum_{i=1}^{n}\sum_{j=1}^{\frac{Nw_{i}}{f}}D_{i}(\vec{r}+{\Delta \vec{r}}_{j}).$$

*D*_*i*_ is determined by shifting the dose opposite the target’s motion. That is to say, when the contour moves in a given direction, from its perspective it sees the dose moving in the contrary direction instead. Thus the target moving posteriorly is equivalent to the beam moving anteriorly, etc. Of course, this relative motion of the dose is not seen by tissues far from the contour—we shall define ‘far’ as distances greater than 1 cm [10] for reasons discussed later—so we restrict our analysis to DVH affected by dose delivered within 1 cm of the prostate. The RT-Plan file is read to note whether the beam would be applied three or four times (*w*_*i*_), and the *n* = 5 beams are summed *Nw*_*i*_/*f* times per beam over *N* = 2000 samples of interfractional motion to maintain this beam weighting. Hence four beams are each applied 375 times with 16/2000 beam dose and one beam is applied 500 times with 16/2000 dose. Thus each beam contributes 2.9 cGy(RBE) to the normalization point thereby summing to the prescribed dose of 57.6 Gy(RBE). *p*(Δ*r*) is therefore approximately some multiple of 1/2000 as determined by the number of times it is randomly sampled. *N* must therefore be sufficiently large to sample the entire probability distribution. Histograms of the sampled distribution verified sufficient sampling.

## Results

**Fig 3:**
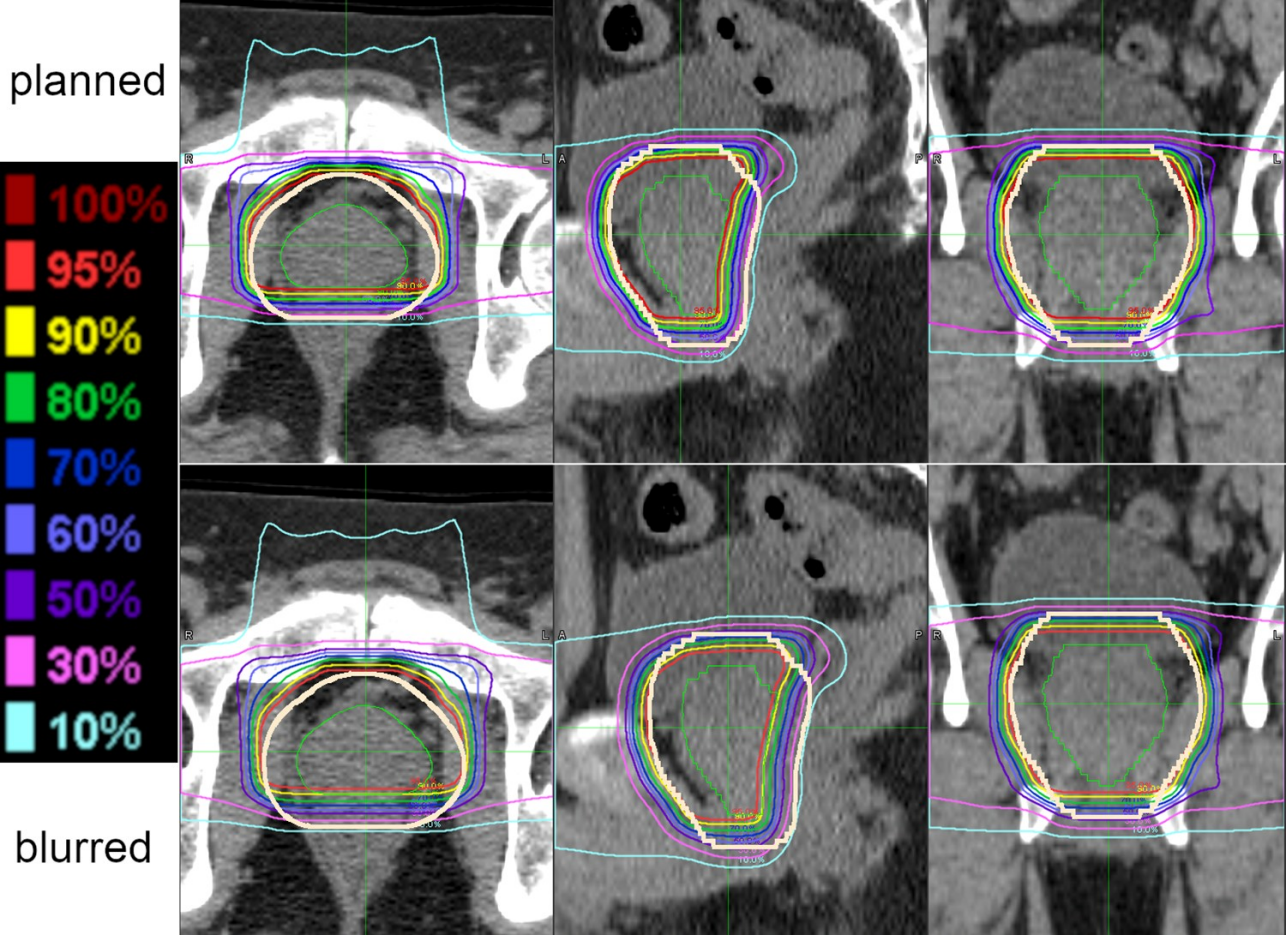
Planned and blurred dose distributions. Planned (upper) and most probable (lower) dose distributions in transverse (left), sagittal (middle), and frontal (right) planes with bold khaki line demarcating a one-centimeter margin around the prostate in which probable dose is reliable.

A comparison of planned and probable dose distributions is made in Fig 3.

**Fig 4:**
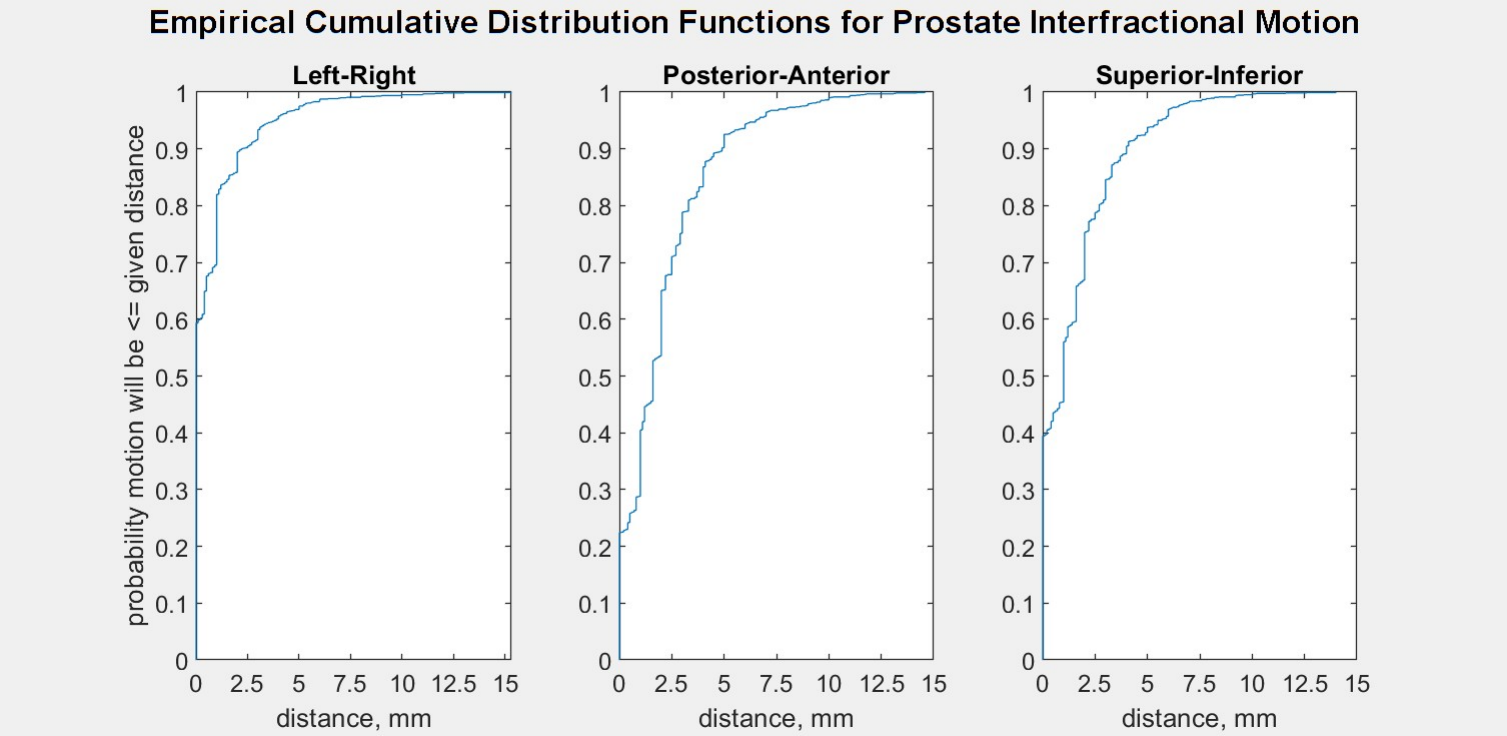
Empirical cumulative distribution function of prostate interfractional distance. Distance from the planning position along each axis.

**Fig 5:**
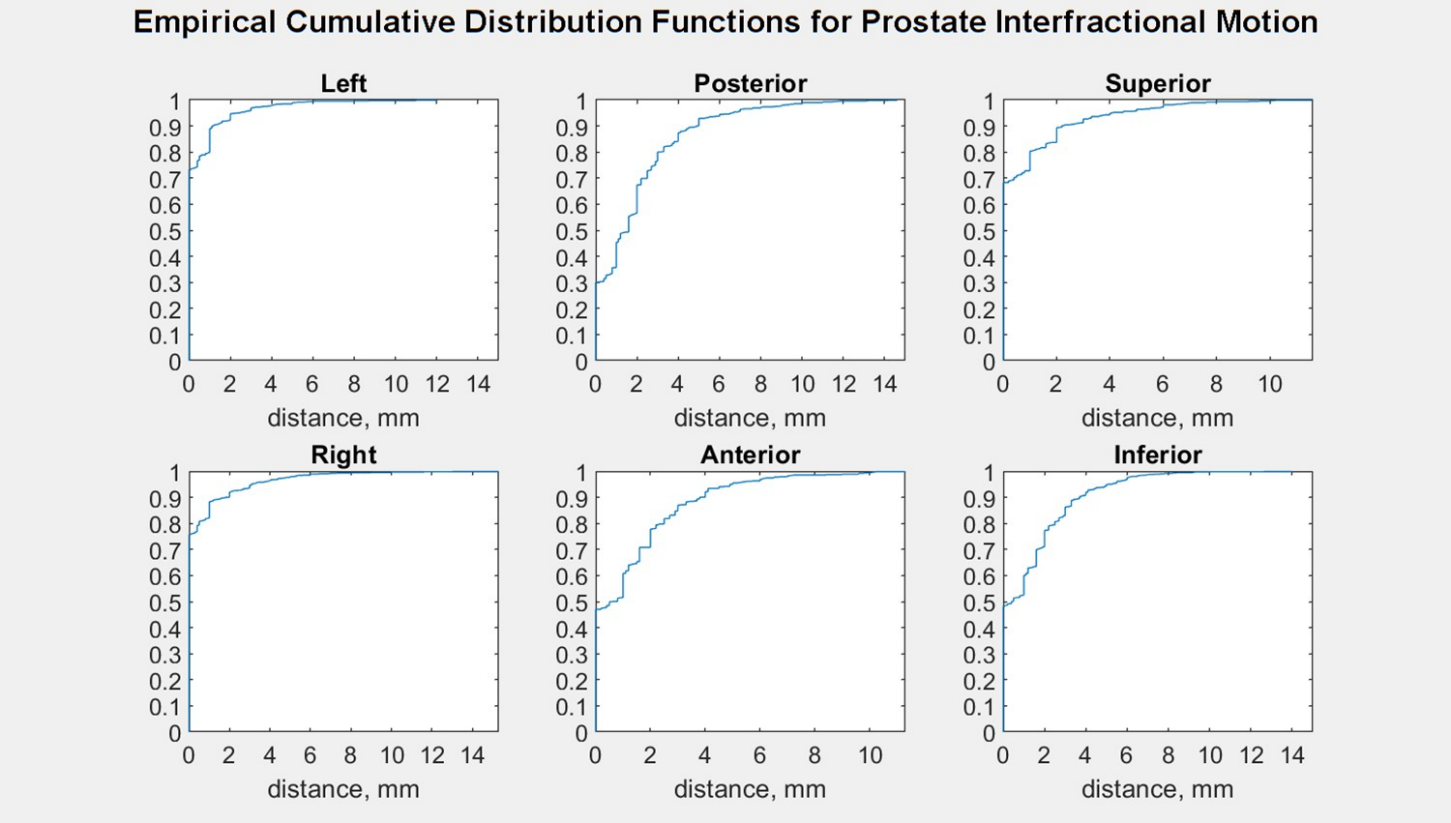
Empirical cumulative distribution function of prostate interfractional displacement. Distance from the planning position per direction.

Empirical cumulative distribution functions of prostate interfractional motion are shown in Figs 4 and 5. Fig 4 shows that for interfractional distance from planned position per DICOM axis, whereas Fig 5 shows that for each direction.

**Fig 6:**
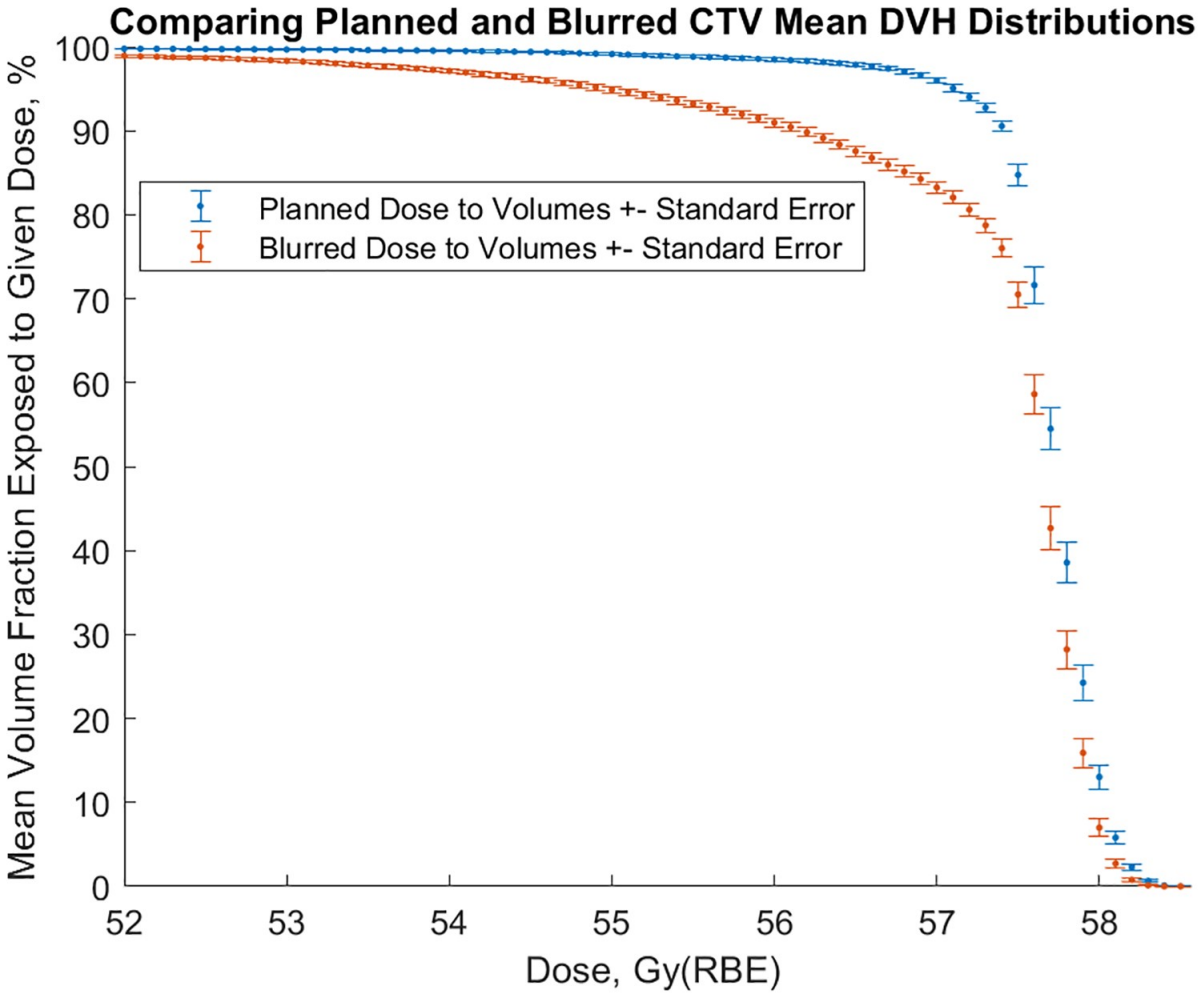
Comparison of mean planned and blurred CTV DVH from 40 patients.

Fig 6 shows mean cumulative DVH for CTV averaged over the patient sample. We note there is essentially no trial overlap, i.e. we should expect this disparity for any 40-patient sample.

**Fig 7:**
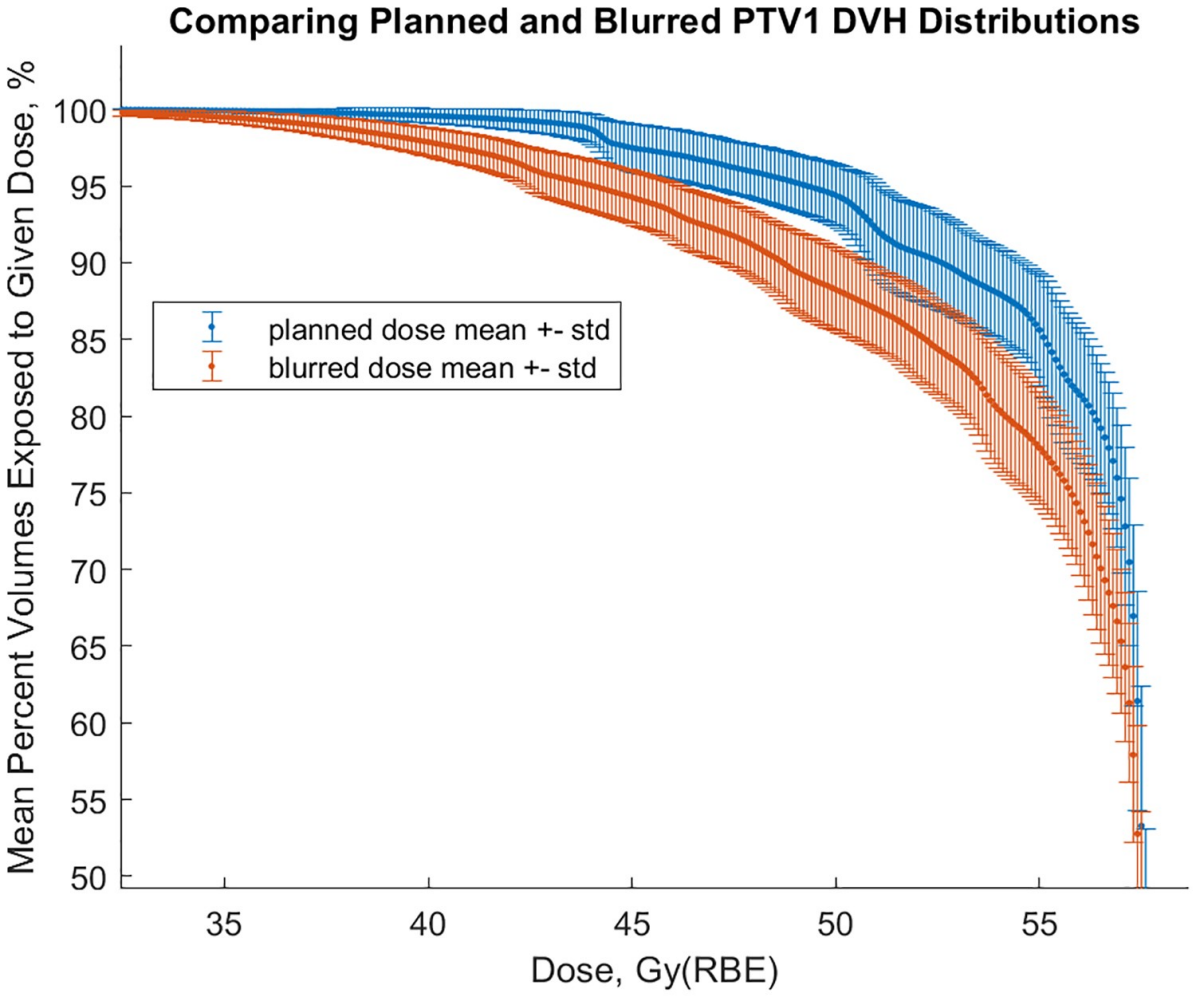
Comparison of mean planned and blurred PTV1 DVH from 40 patients.

Fig 7 shows mean planned and probable DVH for PTV1 volumes averaged over the 40-patient sample. We note that probable dose to the PTV1 is less than planned for nearly every dose level, with a 5–7% drop in coverage.

**Fig 8:**
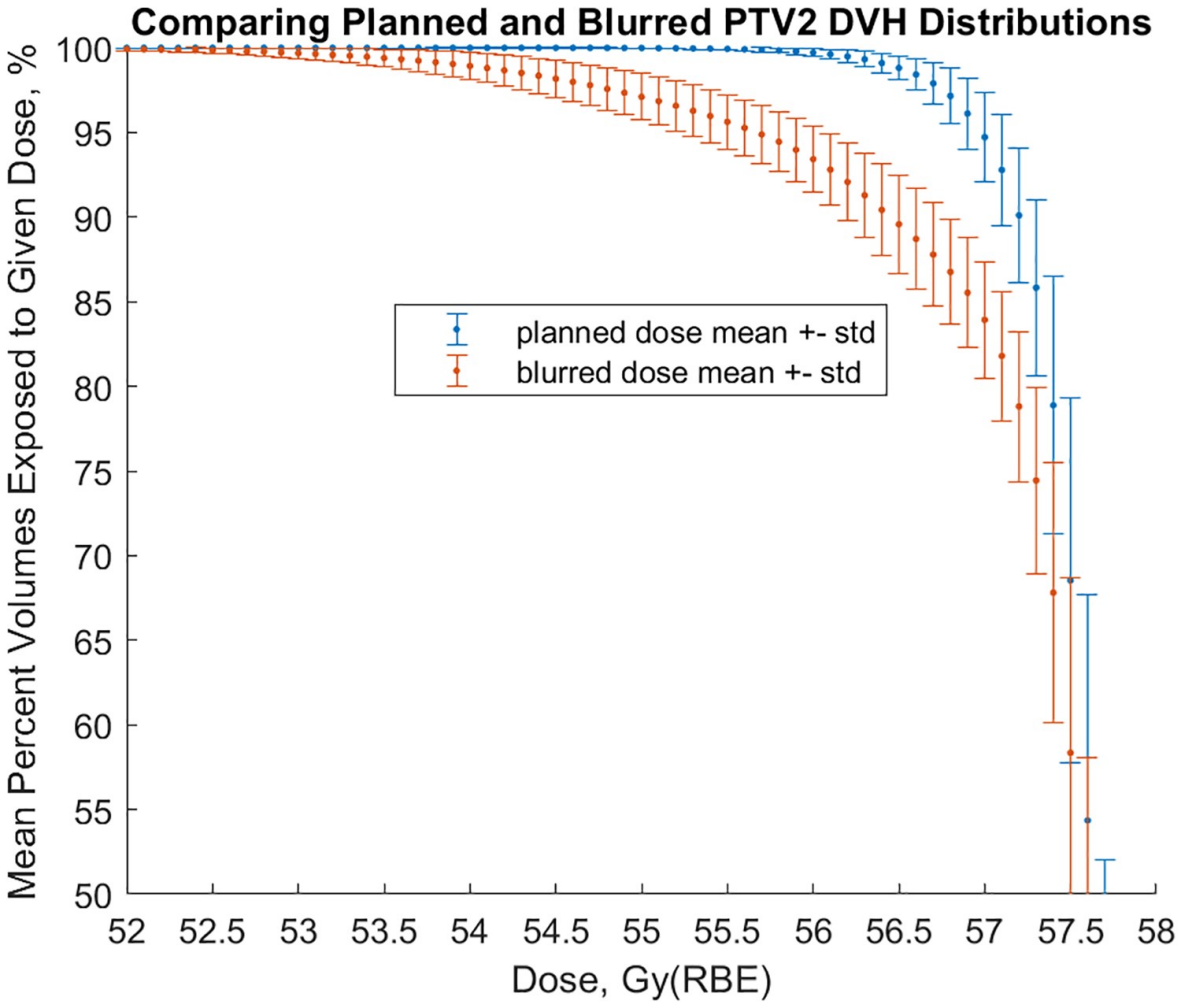
Comparison of mean planned and blurred PTV2 DVH from 40 patients.

Fig 8 shows mean planned and probable DVH for PTV2 volumes averaged over the 40-patient sample. We note that probable dose to the PTV2 is less than planned for nearly every dose level, with about a 10% difference around 57 Gy(RBE).

**Fig 9:**
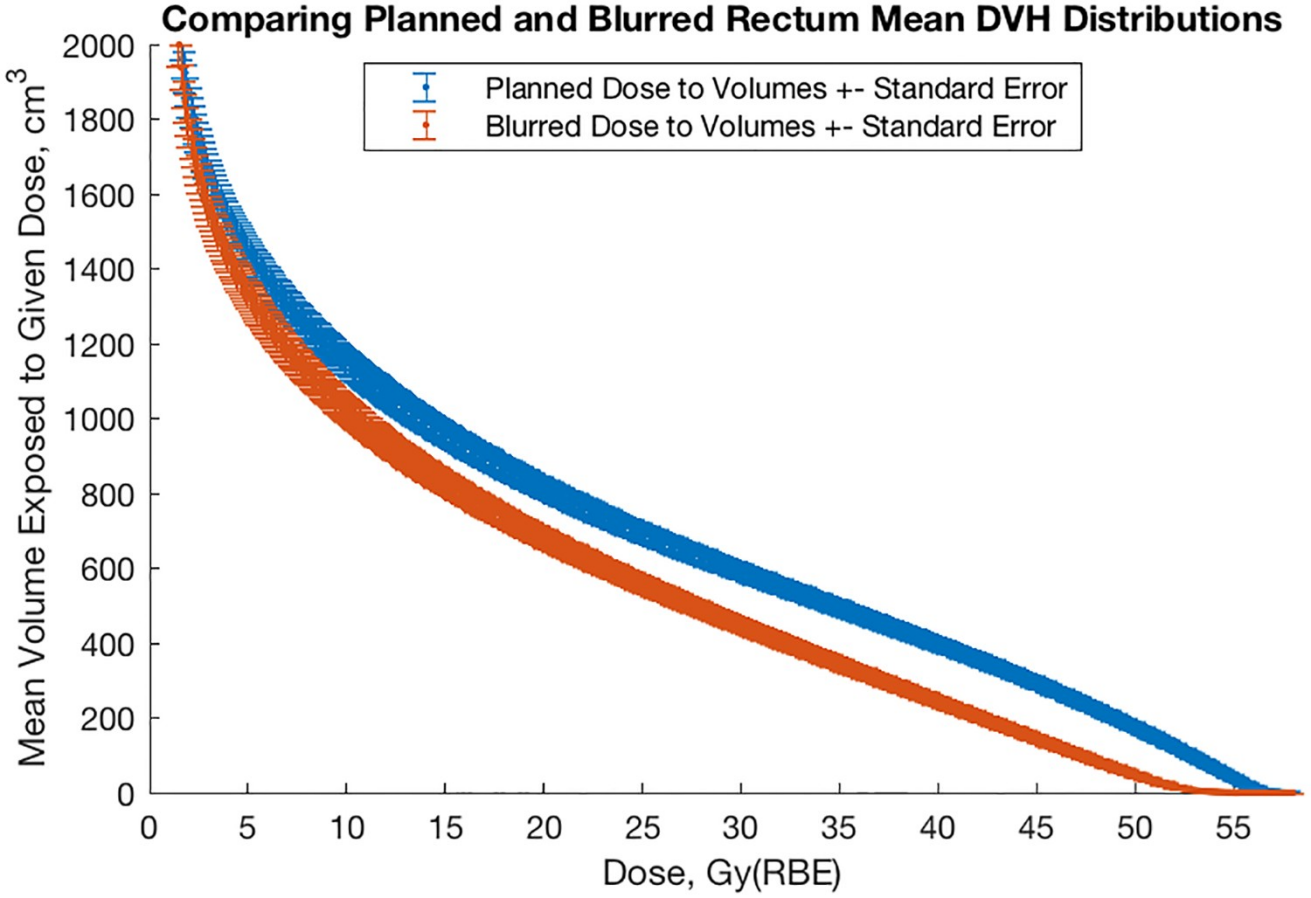
Comparison of mean planned and blurred rectum DVH for 40 CIRT patients.

Fig 9 shows mean planned and probable DVH for physical rectum volumes averaged over the 40-patient sample. We note that probable dose to the rectum is less than planned for nearly every dose level.

**Fig 10:**
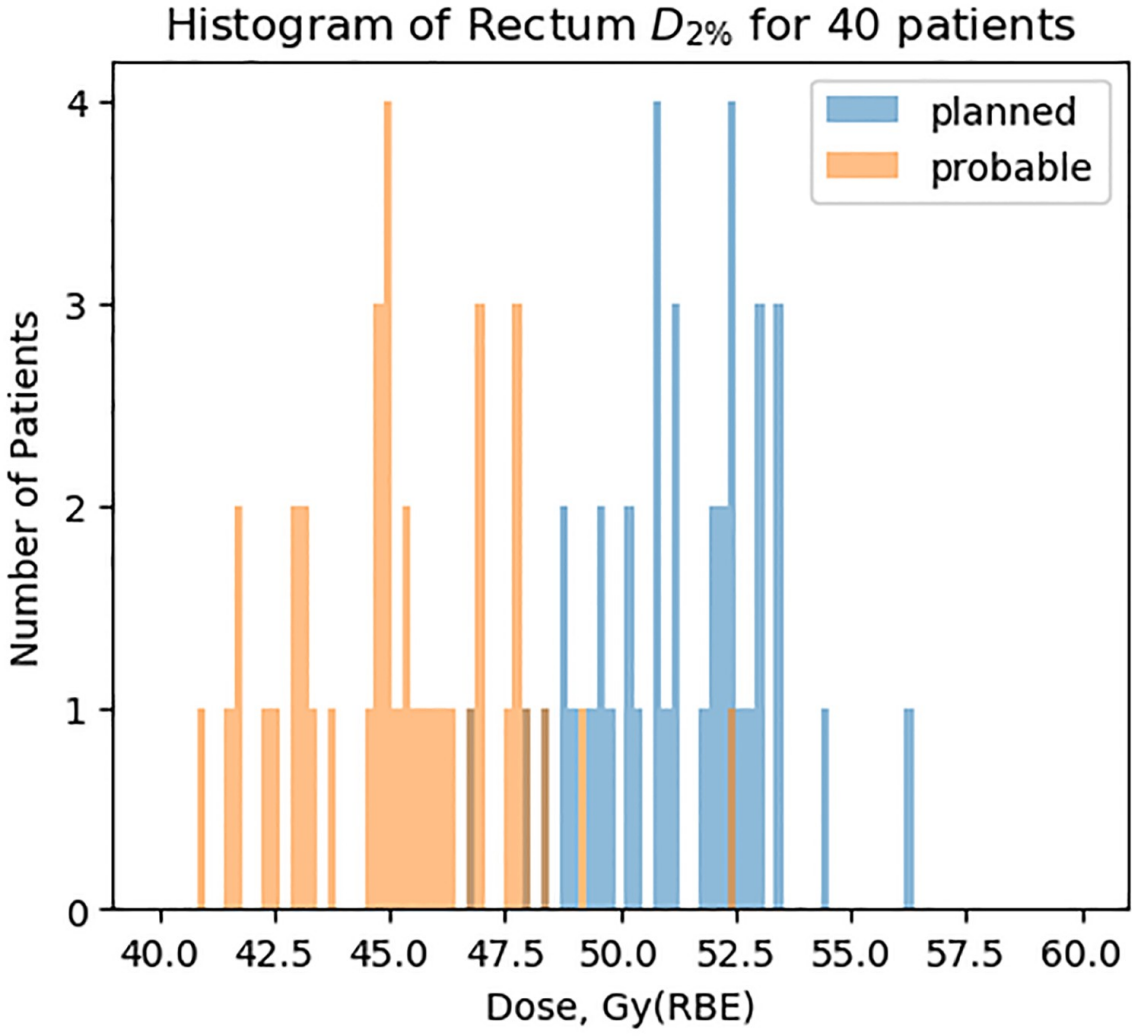
Histograms showing the reduction in D2% resulting from simulated most probable interfractional motion.

Fig 10 shows histograms of D2% comparing planned and blurred dose distributions, revealing a general decrease in dose to rectum as a consequence of interfractional motion.

**Fig 11:**
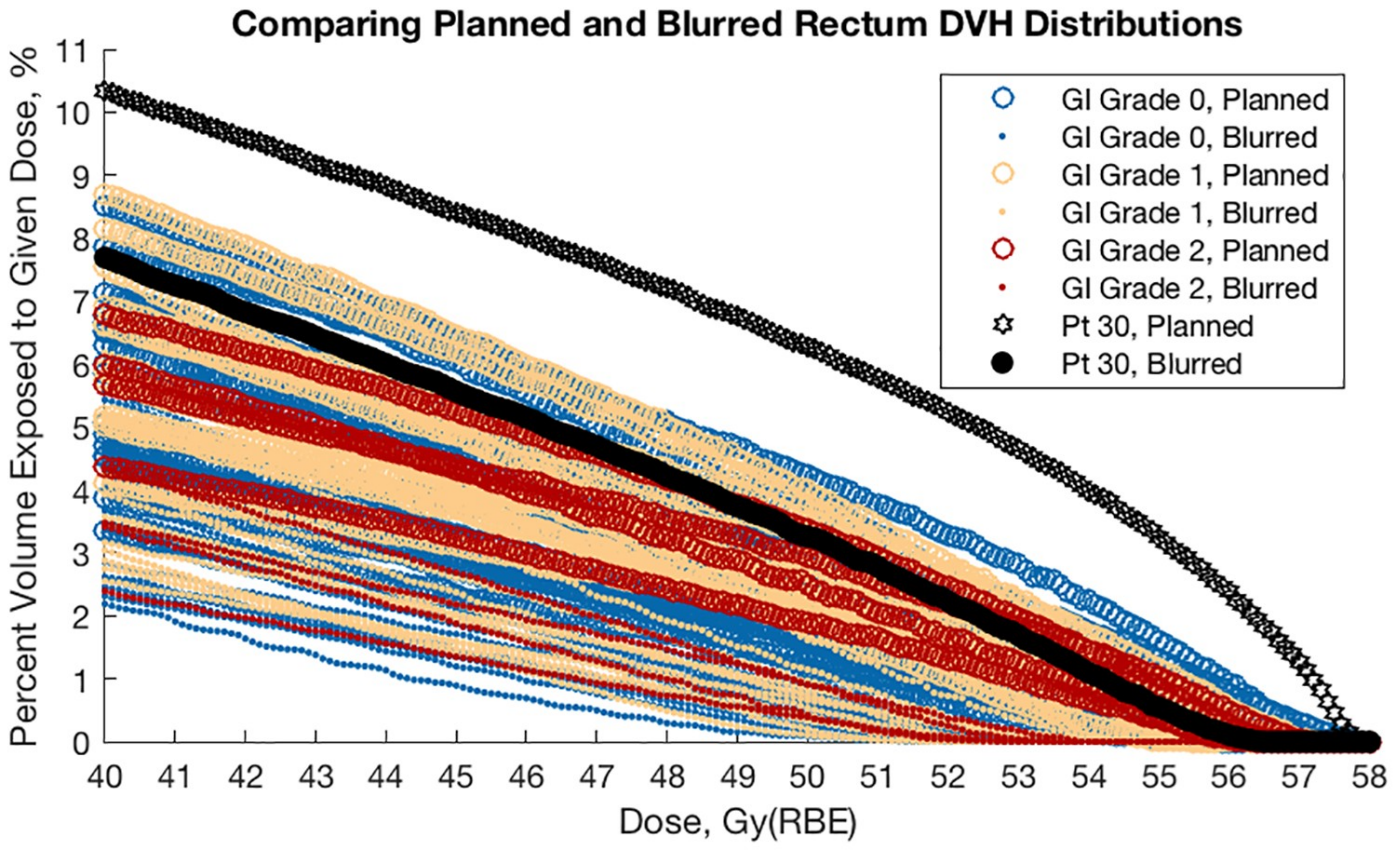
Comparison of patient 30's rectum DVH with the sampled patients reporting gastrointestinal (GI) toxicity. Planned and blurred rectum DVH are color-coded by maximum GI toxicity reported upon follow-up examination.

Fig 11 shows the rectum DVH for the 40 patients sampled, color-coded by toxicity. Special attention is given to the 30^th^ sample, showing a high planned DVH but lower probable DVH. In all cases probable dose is less than planned, however we note here too that GI toxicity is not clearly ordered by rectum size or dose level.

**Fig 12:**
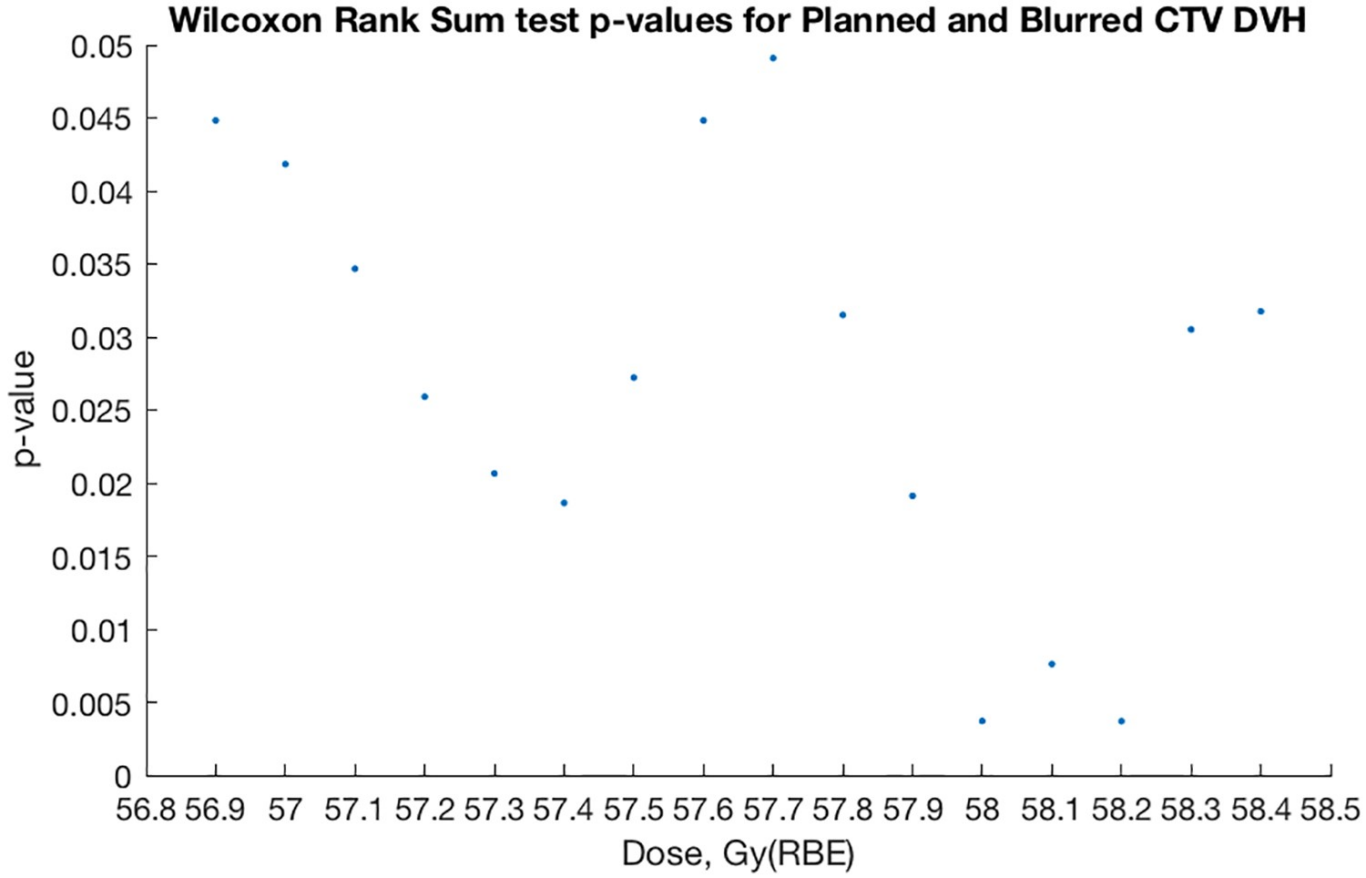
Wilcoxon rank sum test comparing planned and blurred CTV DVH per dose.

Fig 12 shows Wilcoxon rank sum test p-values meeting the 5% significance level indicating statistical difference between planned and probable CTV DVH at the given doses for the 40-patient sample.

**Fig 13:**
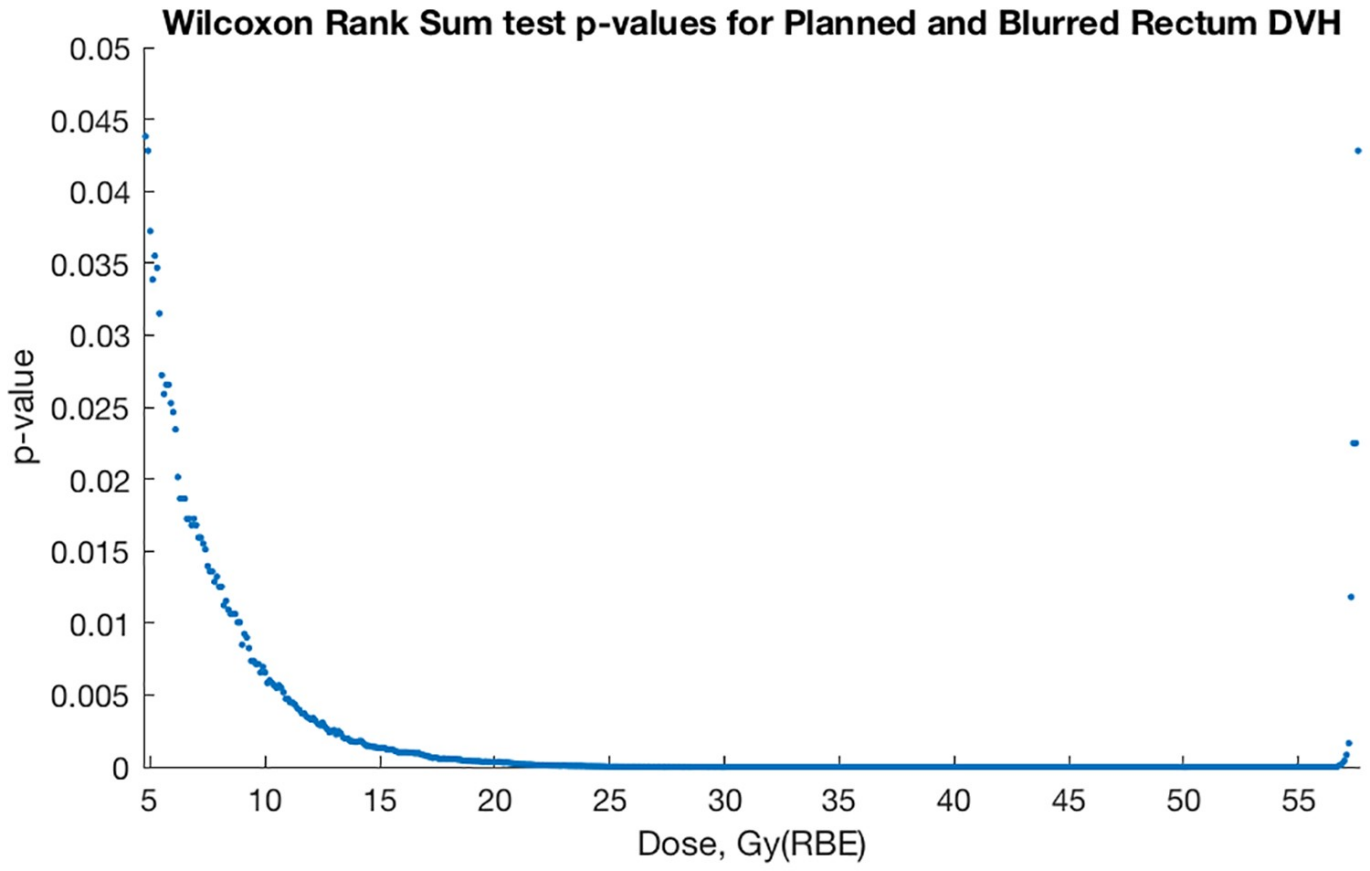
Wilcoxon rank sum test comparing planned and blurred rectum DVH per dose.

Similar to Figs 12 and 13 shows Wilcoxon rank sum test p-values meeting the 5% significance level indicating statistical difference between planned and probable rectum volumes at the given dose for the 40-patient sample. In other words, our 40 samples of rectum volumes and the CTV volumes given a planned dose, when contrasted with corresponding volumes receiving ‘blurred dose’, do not come from continuous parent distributions with equal medians. This is what we mean when we say we have found a statistical difference between planned and blurred dose distributions.

## Discussion

### Justification for static dose assumption given stopping power change

Rigidly shifting the dose distribution to obtain dose within and near the CTV is valid only if range shifts due to changes in stopping power are within established range uncertainty. Bone surfaces have the largest relative stopping power (RSP) to water at around 1.5. By keeping bones in the same position we ensure they do not alter the beam dose as a function of interfractional motion, and hence the dose changes only from soft tissue. With this same foundation Jelen *et al*. have calculated CTV coverage changes due to prostate interfractional motion and have found that the change in dose from stopping power change is negligible for CTV-to-PTV margins greater than 6 mm for shifts up to 10 mm. [10] Moreover, CIRT patients are wrapped in a thermoplastic shell so the change in patient’s surface contour is negligible.

**Fig 14:**
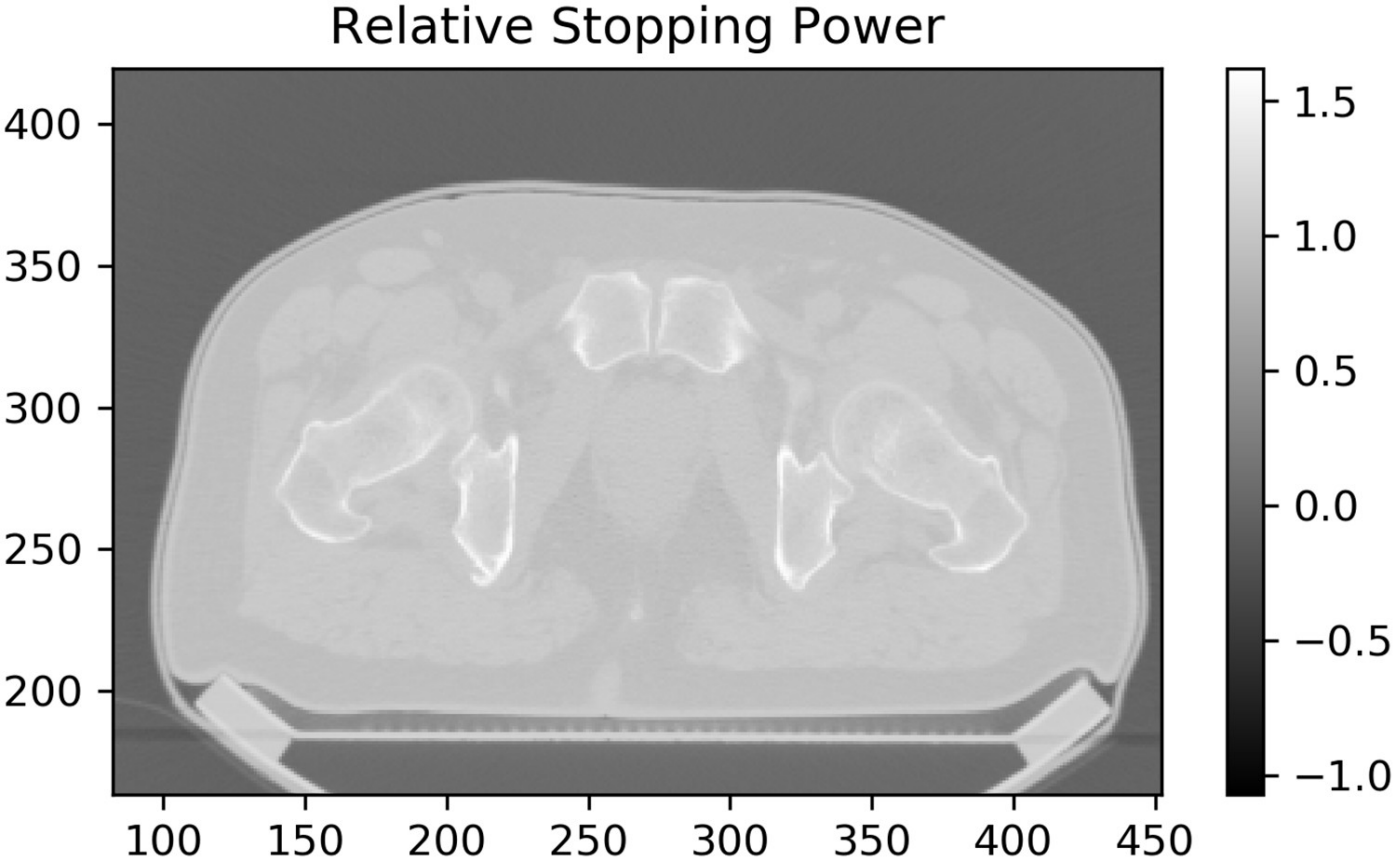
Tissue-to-water stopping power ratio for a typical pelvis CT image. Most of the body has relative stopping power similar to water, with exceptions being bone and hypodense fatty tissue. A thermoplastic shell holds the surface in the same position and bones are realigned to their planned position, so the largest change in stopping power comes from posterior prostate shifts into the anterior region of fatty tissue lateral to the rectum with SPR ~ 0.9.

Fig 14, calculated through linear interpolation from the polybinary tissue model of Kanematsu *et al*. [11], shows stopping power relative to water. Note that most of the body is around 1, comparable to water, with bones being the largest exception and low-density fat lateral to the rectum being the second-most outstanding region. Typical depths of the prostate are 89 mm anteriorly and 184 mm laterally. The prostate shifts 10 mm or less (cf. Figs 4 and 5). The worst-case scenario would be a 1 cm shift posteriorly, introducing about 14 mm of 0.93 RSP rather than 1.04 for a range error of 14 mm x (0.93–1.04) = -1 mm.

However, 1 mm is within the range of uncertainty inherent in the conversion of CT number to stopping power: Paganetti presumes from previous studies that the range uncertainty due to stopping power conversion to be around 0.5%, while “density variations alone might cause an uncertainty below 1%” (p. 5). [12] Taken together, for an anterior depth of 89 mm range uncertainty is already on the order of 1 mm; a lateral depth of 184 mm has a range uncertainty of about 2 mm. Our range error from rigidly shifting the contour (i.e. shifting the dose opposite the contour’s motion to determine dose as seen by the prostate region) is within this inherent uncertainty.

### Statistics and neglected motion

Our statistical test results (Figs 12 and 13) strengthen the theory that planned and probable DVH are of different origins, i.e. that our probable dose-blurring method results in a meaningful difference from planned dose distributions.

Table 1 reports the percent of interfractional motion not covered by our planning margins after considering the empirical cumulative distribution functions of interfractional motion (Figs 4 and 5) with our PTV margins (Fig 2).

**Table 1:** Adequacy of target margins to cover interfractional motion as determined from Fig 5.

Contour	Organ	Direction	Percent of Motion Not Covered, %
PTV1	Prostate	Left	0.18
PTV1	Proximal Seminal Vesicles	Left	3.6
PTV1	Prostate	Right	0.29
PTV1	Proximal Seminal Vesicles	Right	5.3
PTV1	Prostate	Posterior	1.0
PTV1	Proximal Seminal Vesicles	Posterior	9.5
PTV1	Prostate	Anterior	1.0
PTV1	Proximal Seminal Vesicles	Anterior	4.5
PTV1	Prostate	Superior	2.0
PTV1	Proximal Seminal Vesicles	Superior	3.7
PTV1	Prostate	Inferior	3.2
PTV1	Proximal Seminal Vesicles	Inferior	5.2
PTV2	Prostate	Left	0.18
PTV2	Proximal Seminal Vesicles	Left	3.6
PTV2	Prostate	Right	0.29
PTV2	Proximal Seminal Vesicles	Right	5.3
PTV2	Prostate	Posterior	depends on rectum cutline
PTV2	Proximal Seminal Vesicles	Posterior	depends on rectum cutline
PTV2	Prostate	Anterior	1.0
PTV2	Proximal Seminal Vesicles	Anterior	4.5
PTV2	Prostate	Superior	depends on seminal vesicle anatomy
PTV2	Proximal Seminal Vesicles	Superior	depends on seminal vesicle anatomy
PTV2	Prostate	Inferior	48
PTV2	Proximal Seminal Vesicles	Inferior	48

### Moderate CTV degradation

Our mean DVH degradation may seem large at first glance. Our CTV DVH do show about 14% underdosing on average at the prescribed dose, but we consider it moderate loss of coverage for the following reasons. The loss of coverage is to (1) the proximal seminal vesicles, which different clinics treat differently, not clearly diseased, and (2) the posterior prostate, lying adjacent to the rectum, sometimes must receive less dose to spare the rectum. We think this degradation is not a problem in light of our clinical results. We do not feel a sense of urgency that the word ‘large’ suggests and think that ‘moderate’ is more descriptive of the clinical impact of this coverage.

### Usefulness of the blurred dose distribution: CTV analysis

The blurred dose represents the most probable dose distribution that we would expect to see given interfractional motion. If we use this distribution as a second-check, then we can increase the probability of target coverage in the presence of interfractional motion. For example, mean D95 to CTV was 55.1 Gy(RBE) without patient relapse. If we are concerned about a patient’s CTV coverage, we can quickly calculate this blurred DVH. If its D95 is at least 55.1 Gy(RBE), then we may have more confidence in the treatment. We may also consider the probable blurred dose distribution to get an idea what the dose could be without correcting for interfractional motion. Other dose constraints such as minimum dose to PTV and PTV D95 (used for dose prescription) should also be considered.

The blurred dose suggests another dose reduction study may be beneficial. The CTV (i.e. proximal seminal vesicles) might not require currently prescribed dose. Future researchers could use this most probable dose as a starting point for dose prescription reduction: We hypothesize that one can use in-room CT for positioning verification and dose recalculation together with dose verification (e.g. prompt gamma-ray emission [13] or PET autoactivation [14]) to ensure target coverage and reduce D95 to 55.1 Gy(RBE) rather than the planned value of 57.1 Gy(RBE) in the absence of in-room CT. From these use cases we think the blurred DVH most-probable dose method is an important clinical contribution to evaluate carbon ion treatment of targets with uncorrected interfractional motion.

### Limitations of this study

We note that the interfractional motion calculated from photon therapy table shift data is not a normal distribution but rather has central peaks at the origin (Fig 1). We think this discrepancy is caused by the technologist preferring not to shift the patient if the interfractional motion is less than one millimeter, and hence the table shift data has an excessive number of data recorded at the origin.

Moreover, compare the interfractional motion in Fig 1 with the random samples from the fit distributions in Fig 4: By including the central peak in the normal fit, the full-width half-maxima are narrower, implying more motion lies closer to the origin. If we exclude some of the central peak (or divide the histogram count between adjacent counts to assume motion neglected by the technologist) to obtain wider normal distributions, the motion models would have a larger probability of greater shifts. Therefore our model may underestimate interfractional motion, if the central peak is unreliable.

Because we shift the beams rigidly opposing given prostate motion, blurred dose to structures far from the prostate are unreliable. Following the work of Jelen *et al*., [10] we define ‘far’ to be more than 10 millimeters from the prostate. This study only estimates dose to target structures and proximate rectum.

### Gastrointestinal toxicity and blurred rectum DVH

The mean DVH from our patient sample as well as the histograms of D_2%_ indicate that dose to GHMC patients is on average less than planned (Figs 6–12). For example, consider patient 30 of our sample (Fig 11). This patient has had no relapse or rectum toxicity. It appears his planned rectum DVH overestimated the risk of toxicity whereas the interfractional-motion-blurred rectum DVH would have given reassurance to the staff not to sacrifice target coverage. On the other hand, patients with lower planned and blurred DVH experienced worse toxicity, so of course one must not rely solely on rectum DVH, but consider other clinical variables such as isodose curves, prior treatment history, and genetics.

Generally speaking, dose to the rectum is substantially less than planned (Figs 3 and 9–11). This helps explain why the rate of toxicity is so low. [4]

### Planning target volume margins: Photon therapy vs carbon ion therapy

In photon therapy, the standard approach is to define the gross tumor volume as visible disease, prescribe a CTV margin around this location for microscopic disease, and finally calculate a PTV margin around this to account for setup and motion uncertainty. In IMXT, CTV may be defined as the prostate including suspected growth and the proximal 1–2 cm of seminal vesicles. [15] PTV1 extends the CTV by 3 mm in all directions. PTV2 extends the CTV by 15 mm in all directions. Alternatively, in image-guided photon radiotherapy PTV may be extended from the prostatic CTV 2, 8, and 6 millimeters in the right-left, posterior-anterior, superior-inferior directions respectively; seminal vesicle margins may be 4, 10, and 9 millimeters, respectively. [16] Another option for volumetric-modulated arc photon therapy margins is to define the CTV as the prostate with proximal 1.5 cm seminal vesicles, and PTV extending this by 8 mm in all directions except posteriorly, extending by 5 mm in this direction. [2] Of course, from a theoretical perspective, CTV should not depend on radiation modality, but rather the spread of disease.

In carbon ion radiotherapy, the approach to planning target volume differs due to water equivalent path length (WEPL) dependence. [14] In photon therapy, margins are added mostly for change in location (setup position, target motion). In particle therapy, targeting must additionally account for beam depth, i.e. the WEPL seen by the carbon ion from the patient surface to the distal end of the target. This depth is a function of the stopping power of tissues penetrated along the beam, and therefore depends on the patient’s anatomy rather than a fixed formula. However, patients treated with the protocols considered by this study had CTV and PTV as described above in the Methods subsection “16-fraction Prostate Cancer Therapy at the Gunma University Heavy Ion Medical Center”. We note that these carbon radiotherapy planning target volumes are similar in size to those in photon radiotherapy.

### Suitability of PTV margins

Our photon therapy table shift data analysis indicates interfractional motion larger than 3.8, 6.5, and 5.5 mm in each DICOM axis occurs in 5% of patient setups (Figs 1 and 4). To describe this motion in other terms, the chance of the prostate being shifted more than 3 mm from the planned position is 7%, 22%, and 17% in each DICOM axis. Comparing our PTV margins (Fig 2) with this observed motion via empirical cumulative distribution functions (Fig 5), their adequacy in covering interfractional motion is tabulated in Table 1. At first glance it would seem good to have more margin around proximal seminal vesicles and inferior prostate for PTV2. However, such expansion is difficult due to the proximity of the rectum. Moreover, given our clinical results, the disease appears sufficiently treated already. So overall our margins appear suitable for our patient population.

This dose blurring method rigidly shifts the dose distribution, so when the prostate falls posteriorly the seminal vesicles are assumed to do so as well, moving further out of the beam. For this reason blurred CTV DVH are less than planned (Fig 6). Nonetheless, our rate of recurrence is very low. Only one of the 40 patients sampled had PSA relapse, and this fraction (2.5%) is higher than the clinical population because we deliberately biased our sample to overrepresent patients with toxicity.

Given the very good therapeutic results of CIRT with the current bone-matching method, [4,5] our results imply two possibilities, namely, the target may be smaller and less prescribed dose may be needed at the GHMC. More investigation would be useful to clarify which of these implications is correct.

### Dose calculations using CBCT and in-room CT contrasted with our blurred dose

The reader may ask, “Why estimate most likely dose distribution rather than recalculate the dose based on CBCT or in-room CT?” The short answer is that we did not have CBCT or in-room CT when this study began, and consequently this study looks retrospectively at prior clinical practice with intended application for centers who haven’t yet adopted such methods. A more detailed examination of such methodology follows.

In 2007 Yang et al. found IMXT and 3D conformal photon radiotherapy dose calculations between CBCT and planning CT to differ by roughly 2%. [17] This was good development for photon radiotherapy, but still not precise enough for charged particles, as sharp distal fall-off introduces more uncertainty in CT number. [18] More recently, earlier this year Marchant *et al*. produce CBCT-based photon therapy treatment plans differing from planning CT-based plans by less than 1%. [19] Yet their shading correction algorithm holistically blurs CT numbers using a linear scaling derived from histograms, overrides heterogeneities through surrounding soft-tissue interpolation, and overrides bone with surrounding soft-tissue to create a correction map applied to alter the CBCT image (p. 2). This holistic altering of the image thereby presupposes beams traversing it in a holistic manner, as indeed their IMRT, VMAT, and 3DCRT photon beams do. It appears in this way errors from heterogeneous regions are smoothed out overall. This method blurring over heterogeneities cannot be applied to charged particle therapy since it is precisely these heterogeneities that matter the most as traversed tissues stop the beam, as particle range and dose is much more sensitive to tissue inhomogeneity than photons. Moreover, they note cases where image deformation fails (p. 6) or introduces errors (p. 5) which would likewise amplify error in charged particle therapy. Because inaccurate Hounsfield numbers in CBCT presents a problem for treatment planning, [20] we prefer in-room CT for adaptive radiotherapy. It is beyond the scope of this study to conduct a literature review on CBCT innovation, but Marchant *et al*. introduce the interested reader to numerous recent studies. [19] Van Elmpt and Landry introduce the interested reader to recent work on CBCT-based dose calculation for proton therapy. [21] We look forward to seeing CBCT-based calculation methods continue to improve as personalized treatment planning increases precision of care.

## Conclusion

Given probable loss of CTV coverage already evident at 16 fractions (Fig 6), it is dangerous to further decrease fractionation with bone-matching without accounting for interfractional motion: It is imperative to confirm CTV position relative to heterogeneities in the beam path prior to hypofractionated beam delivery, and modulate the beam energy if the prostate falls posteriorly. In-room CT can be used for this purpose: Once CTV location is confirmed the treatment may be replanned, e.g. for minor shifts using the method of Jelen *et al*. [10] We may also verify how deep the rectum lies beyond the Bragg peak, a critical detail for hypofractionation. However, CT analysis and any adaptive replanning must be done quickly; otherwise the CIRT benefit of a one-minute treatment time is lost and intrafractional motion must be considered.

As stated previously, the clinical target volume may be smaller than expected or less dose may be needed than currently planned. In-room CT may prove useful together with PET autoactivation [14] or developing methods with prompt-gamma imaging [22] to ascertain *a posteriori* the target size based on long-term patient followup. Of course, use of CT also increases dose to the patient: Roughly 1 Gy(RBE) is delivered to the rectum per beam during a typical treatment (cf. Figs 9–11), whereas a typical CT scan delivers roughly 3.10 mSv. [23] Being roughly 300 times smaller than the dose from a typical CIRT fraction, we may use in-room CT without altering our planned dose, though the principle of ‘as low as reasonably achievable’ applies nonetheless.

We recommend carbon ion radiotherapy clinics without in-room CT use our probable blurred dose method to consider a fuller picture of the dose probably being delivered to the patient given only bone-matching positioning. We recommend clinics with in-room CT to use our probable blurred dose method to establish a baseline for newly-adjusted DVH analysis during treatment planning consultations.
